# Is Life Unique?

**DOI:** 10.3390/life2010106

**Published:** 2011-12-30

**Authors:** David L. Abel

**Affiliations:** Department of ProtoBioCybernetics and ProtoBioSemiotics, Origin of Life Science Foundation, Inc., 113-120 Hedgewood Drive, Greenbelt, MD 20770, USA; E-Mail: life@us.net; Tel.: +1-301-441-2923; Fax: +1-301-441-8135

**Keywords:** formalism, prescriptive information (PI), sustained functional systems (SFS), functional sequence complexity (FSC), the law of organizational and cybernetic decline (The OCD Law), the formalism > physicality (F > P) principle, choice-contingent causation and control (CCCC), the cybernetic cut, the configurable switch (CS) bridge, the organization (O) principle

## Abstract

Is life physicochemically unique? No. Is life unique? Yes. Life manifests innumerable formalisms that cannot be generated or explained by physicodynamics alone. Life pursues thousands of biofunctional goals, not the least of which is staying alive. Neither physicodynamics, nor evolution, pursue goals. Life is largely directed by linear digital programming and by the Prescriptive Information (PI) instantiated particularly into physicodynamically indeterminate nucleotide sequencing. Epigenomic controls only compound the sophistication of these formalisms. Life employs representationalism through the use of symbol systems. Life manifests autonomy, homeostasis far from equilibrium in the harshest of environments, positive and negative feedback mechanisms, prevention and correction of its own errors, and organization of its components into Sustained Functional Systems (SFS). Chance and necessity—heat agitation and the cause-and-effect determinism of nature’s orderliness—cannot spawn formalisms such as mathematics, language, symbol systems, coding, decoding, logic, organization (not to be confused with mere self-ordering), integration of circuits, computational success, and the pursuit of functionality. All of these characteristics of life are formal, not physical.

## 1. Introduction

Multiple peer-reviewed publications have appeared in the literature that have asked the question, “What is life?” Those authors who have attempted to provide a definition have typically experienced a great deal of frustration. In 1996, Rizotti published a book, *Defining Life: The Central Problem in Theoretical Biology* [1]. Rizotti collected and compared many attempted definitions of life from the literature. No one definition seemed to be accurate, succinct, and adequate.

The community of life-origin investigators (ISSOL, the International Society for the Study of the Origin of Life, now the Astrobiology Society) has always had an acute interest in defining life. NASA’s definition of life, usually attributed to Gerald Joyce of the Scripps Institute [2], says merely that “Life is a self-sustaining system capable of Darwinian evolution.” This definition, however, is painfully naïve, simplistic, and wholly inadequate for biological research. Many who formally subscribed to this definition of life have since added additional requirements to the definition. The most common addition has been the requirement of a primitive membrane [3,4,5,6,7,8,9,10,11,12,13,14,15]. Many others feel that minimal independent life must also manifest a crude homeostatic metabolism [16,17,18,19,20,21]. Others feel that a hereditary symbol system that can mutate independent of its phenotypic realization is essential (necessary for open-ended evolution [22,23,24,25,26,27,28,29,30]). Many others affirm the reality and need not just for Shannon uncertainty, but for literal positive genetic and epigenetic instructions [25,31,32,33,34,35,36,37,38,39,40,41,42,43,44,45,46]. Greisemer ([47], p. 35) points out that epigenetic modifications to genetic material all count as hereditary changes in the broadest sense [48,49].

To be fair, Joyce’s definition was never intended to be empirically responsible in describing everything we observe about current life. It was designed, instead, to make our naturalistic life-origin models seem more plausible. Unfortunately, defining down life to make our life-origin theories “work for us” has little to do with unraveling what is *objective* life.

At the turn of the millennium an international conference of University Professors was called in an attempt to collectively decide, “What is life.”[50] Every participant was required to submit a definition of life in advance. Every speaker, of which I was one, was required to address the question. No two definitions of life were the same. In the anthology that grew out of the conference, *Fundamentals of Life* [51], the contest of ideas continued on with no clear resolution of what constituted life from a purely scientific perspective. It was interesting at that time, however, that all of the definitions presented could be divided into two subsets: one subset contained biophysicist Hubert P. Yockey’s notable definition; the other subset containing everybody else’s definitions! Yockey made the unique observation that “there is nothing in the physico-chemical world (apart from life) that remotely resembles reactions being determined by a sequence and codes between sequences. The existence of a genome and the genetic code divides living organisms from non-living matter” [52,53].

Yockey [54,55,56,57,58,59] was among the first to realize the linear digital nature of genetic control. Many others have appreciated that life was somehow different, but could not put their finger on exactly what this difference is. Ernst Mayr [60,61] argued that physics and chemistry do not explain life. Monod [62] and Bohr [63] argued the same. Bohr pointed out, “Life is consistent with, but undecidable from physics and chemistry.” Küppers agreed [64].

Mark Bedau calls attention to the Program-Metabolism-Container (PMC) model of life origin [65]. This approach attempts to reduce life to a functionally integrated triad of chemical systems. The model fails, unfortunately, to recognize the formal nature of “Program” in the triad, especially when arbitrary symbol systems such as the codon table are used to represent instructions and controls. By arbitrary, we do not mean random, but rather physicodynamically indeterminate assignments that can only be considered formal rather than physicochemically caused. A large number of symbol systems besides the codon table are employed by life, as Barbieri and many other biosemioticians have pointed out in multiple publications [40,66,67,68,69,70,71,72,73,74,75,76,77]. Donald E. Johnson addresses what might be a minimal genome in the first protocell [78].

In the new millenium, the dichotimization of life from non-life has become far more specific and clear as a result of the relatively new scientific disciple known as ProtoBioCybernetics [79,80,81]. Cybernetics studies “control”. “ProtoBio” refers to “primordial ‘life’”. The most fundamental distinction is *the ability of “life” to exercise formal (nonphysical) organizational and pragmatic control* over its otherwise physical interactions (e.g., chemical reactions, molecular associations, electrostatic attractions/repulsions; hydrophilic/hydrophobic tendencies, phase transitions; quantum uncertainty and “information entanglement”) [82,83,84]*.* The formal controls are attributable specifically to Prescriptive Information (PI) [79,80,81,82,85,86] and its carefully regulated algorithmic processing. More than anything else, the ability to organize, regulate and holistically manage physicodynamics into a formal meta-metabolic scheme that values and pursues staying alive is what defines the uniqueness of life [87,88].

Carol Cleland at the University of Colorado warns against the relentless pursuit of life’s definition [89], pointing to the limitations of language. Also crucial in any attempted definition of life is the influence of prior presuppositional (metaphysical) commitments that we bring with us to science. These philosophic pre-assumptions color what one is willing to acknowledge of the formal organization and controls in molecular biology that we repeatedly observe.

Therefore, in this inaugural issue of a very welcome new MDPI-quality periordical, *LIFE*, we choose instead to ask the question, “What is unique about life?”

- What are some of the criteria that allow us to distinguish life from non-life?
- Can life spontaneously generate from physicodynamic interactions alone?
- Will our intelligence ever be able artificially to construct life from inanimate chemical components?
- What exactly does it mean for life to die?

## 2. The Simplest Known Free-Living Organism

Reductionism has served science well. In biology, however, cellular vivisection can easily destroy the very holistic cellular life that we set out to investigate [41,90,91,92,93,94].

Tibor Ganti, a prominent life-origin theorist, argues, “The basic units of theoretical biology must be sought at the organization level of prokaryotes.” ([47], p. 62) The simplest prokaryotes known are the mycoplasmas and thermoplasmas. *Carsonella ruddii* is not free-living. The simplest known autonomously replicating organism, *Mycoplasma genitalium,* has a 580-kilo-base-pair genome containing 470 genes [95]. Few of these genes seem to encode conventional transcription regulators [94,96,97,98,99,100,101,102,103]. Evidence now exists that changes in DNA supercoiling can regulate transcription in *Mycoplamsa genitalium* [95]. The number of interacting, formally integrated layers and dimensions of life’s Prescriptive Information (PI) [42,79,80,81,82,83,85,86,104], even in the simplest known free-living organisms, is mind-boggling.

## 3. The Invariant Characteristics of Life

Perhaps a good place to start is a descriptive list of free-living life’s invariant characteristics. Sustained, free-living “life” is any system which from its own inherent set of biological instructions and algorithmic processing of that Prescriptive Information (PI) can perform all nine of the following biofunctions (Used with permission:[105]):

(1) Delineate itself from its environment through the production and maintenance of a membrane equivalent. In theoretical early life, this membrane equivalent would most likely have been a rudimentary or quasi-active-transport membrane necessary for selective absorption of nutrients, excretion of wastes, and overcoming osmotic and toxic gradients,
(2) Write, store, and pass along into progeny Prescriptive Information (PI) (instructions, both genetic and epigenetic/epigenomic) needed for organization; provide instructions for energy derivation and for needed metabolite production and function; symbolically encode and communicate functional message through a transmission channel to a receiver/decoder/destination/effector mechanism; integrate past, present and future time into its biological PI (instruction) content (PI instructions can be implemented now or any time in the future. In addition, according to evolution theory, these instructions embody a protracted history of derivation and former control. PI is thus largely time-independent, a feature that bespeaks its formal rather than physical essence.),
(3) Bring to pass the above recipe instructions into the production or acquisition of actual catalysts, coenzymes, cofactors, *etc*.; physically orchestrate the biochemical processes/pathways of metabolic reality; manufacture and maintain physical cellular architecture; establish and operate a semiotic system using “signal molecules”,
(4) Capture, transduce, store, and call up energy for utilization (intuitive, useful work),
(5) Actively self-replicate and eventually reproduce, not just passively polymerize or crystallize; pass along the apparatus and “know-how” for homeostatic metabolism and reproduction into progeny,
(6) Self-monitor and repair its constantly deteriorating physical matrix of bioinstruction retention/transmission, and of architecture,
(7) Develop and grow from immaturity to reproductive maturity,
(8) Productively react to environmental stimuli. Respond in an efficacious manner that is supportive of survival, development, growth, and reproduction, and
(9) Possess enough relative genetic stability, yet sufficient mutability and diversity, to allow for adaptation and potential evolution.

All free-living classes of archaea, eubacteria, and eukaryotes meet *all* nine of the above criteria. Eliminate any one of the above nine requirements, and it remains to be demonstrated whether that system could remain “alive”. RNA strands, DNA strands, prions, viroids, and viruses are not free-living organisms. They fail to meet many of the above well-recognized characteristics of independent “life”.

Even in historical and theoretical science, there must be some degree of empirical accountability to our theories. Models of life origin *must not consist of “defining down” the meaning and essence of the observable phenomenon of “life” to include “nonlife” in order to make our theories “work for us”*. Any scientific life-origins theory must connect with “life” as we observe it (the “continuity principle”). Science will never be able to abandon its empirical roots in favor of purely theoretical conjecture. On the other hand, science must constantly guard itself against Kuhnian paradigm ruts. We must be open-minded to the possibility that life has not always taken the form that we currently observe. In addition, we must take into consideration the problems inherent in any historical science where the observation of past realities is impossible.

## 4. Life Is Formally Controlled, Not Just Physicodynamically Constrained

Constraints and controls should never be confused [106]. Constraints consist of initial conditions, boundary conditions, and the law-described orderliness of nature itself. Controls are always formal. Controls steer events toward potential function and bona fide organization (not just low-informational self-ordering) [107].

No single word relative to biological investigation in the last five years has dominated the scene more than the word, “regulation”. All known life is cybernetic. “Cybernetic” means steered, controlled and/or regulated with purposeful intent. What naturalistic life-origin science has never been able to explain is how inanimate physicochemistry could have formally integrated components and circuits into such holistic organization. Reactions are guided through pathways and cycles into highly conceptual, abstract, functional metasystems. Sophisticated components are manufactured and assembled into molecular machines and nanocomputers [108] that all cooperate to achieve goals normally considered to be formally transcendent to mere physical interactions. Life is a programmed and pragmatic enterprise. Mere physicodynamic constraints have no motives. They have no formal agendas. They do not pursue functional success.

Programming requires making formal choices at decision nodes. Those decisions can be represented by symbols (e.g., 0 *vs.* 1 to represent a binary choice; or A, G, T or C to represent a quaternary d-bit {dual bit} choice). Symbols can represent physical symbol vehicles (tokens such as Scrabble pieces or nucleotides). Computer programming is about creating semantic constructs that can be translated/compiled to run on a given computational system. The system could just as easily be quaternary rather than binary, as is the case with DNA base-4 prescription of biofunction.

Most of life’s formal controls are represented by a linear digital Material Symbol System (MSS) [25,83,85,106]. The term MSS was first used by Rocha in his Ph.D. thesis [25,109]. Recorded signs, symbols and tokens outside of human minds are representational physical entities called “physical symbol vehicles (tokens)”. Any system of communication using these physical symbol vehicles is a material symbol system. The pressing question becomes, “How can a physical symbol vehicle, or syntax of such physical symbol vehicles in a MSS, *represent* instructions in a purely materialistic world?” [110,111,112,113,114] Neither instructions nor their representation with symbols can be generated by chance and/or necessity. Only abstract, conceptual formalisms, not physicality, can address such questions.

The problem of formalism includes the measurement problem not only in quantum physics, but in Newtonian physics as well. As physicist Howard Pattee has pointed out in many publications, the measurements of initial conditions used in the laws of physics are formal representations (mathematical symbols) of physicality, not physicality itself [113]. The same is true of the laws of physics themselves. They are mathematical constructs. The purely metaphysical belief system known as materialism, upon which naturalism is largely based, cannot explain the existence or role of such formalisms. Language, logic theory, computation, and computer programming are all formalisms. Even science, an epistemological (knowledge) system of mind, is itself a purely formal enterprise. Neurophysiology has not done well trying to reduce the formalisms of consciousness to physicality alone. Clearly, the observed properties of life at all levels require the acknowledgement of formal controls that cannot be explained by chance and/or necessity.

Life uses molecular physical symbol vehicles (tokens: e.g., nucleotides) to “speak” and send meaningful (biofunctional) messages through the cell, between cells, and between organ systems. These tokens are selected from an alphabet of tokens. We can say naively that the alphabet consists of four possibilities. In biochemical reality, the alphabet consists of a lot more than four options. Other nucleotides than adenine, guanine, thymine and cytosine exist that could be polymerized (e.g., pseudouridine, dihydrourine, inosine, 7-methylguanosine, hypoxanthine, xanthine, 2,6-diaminopurine, 6,8-diaminopurine). Nucleotides with left-handed sugars exist could have been polymerized in a prebiotic environment. The exclusion of options other than the big four nucleobases in DNA or RNA is itself a form of life’s formal control mechanisms. Hereditary cytosine and occasionally adenosine methylations alone probably extends the material symbol system to base-5 rather than base-4. However, the polymerization of each non-templated nucleotide in a positive informational strand of DNA naively represents a pragmatic quaternary programming choice. Each polymerization constitutes a quaternary decision node choice. It is a dual-bit logic gate. A nucleotide polymerization can also serve as a configurable switch setting that serves to integrate circuits. These programming realities are ultimately formal, not physical, even though physical symbol vehicles are used in the symbol system (a MSS). Symbol systems are unique to life. So is linear digital instructions and Prescription Information (PI). Noncoding regulatory microRNAs, mRNA editing, post-translational editing, DNA methylations and other epigenetic controls all demonstrate many additional undeniable formalisms.

## 5. Life Is Largely “Computed” by Algorithmic Processing of Linear Digital Programming

Linear refers to a uni-dimensional, sequential string of representational command characters. The simplest computer programming, for example, is directed by such a linear digital string of purposeful binary choice commands represented by either a “1” or a “0”. The sequencing or syntax of these choice-contingent commands provides a growing hierarchy of computational functionality.

Digital means each unit is discrete and definite. Programming choices have an “excluded middle”. Switches must be turned either on or off. There is no in-between. A definiteness and clarity exists with each chosen command. No gray zone exists. Each selection is black or white.

Three-dimensional genomes have been suggested in theoretical protolife (e.g., crystalline genes [115] and composomes [7,116,117,118]). None of these models, however, has fared well through time. All known life depends upon linear digital prescriptive information and cybernetic programming. Even most epigenetic factors are instructed and “manufactured” via transcription, editing and translation.

The place to begin understanding the phenomenon of linear digital prescription is a study of the three different types of sequence complexity [42,83,119]. Biologically functional linear complexity lies in the subset of Functional Sequence Complexity (FSC), not Ordered Sequence Complexity (OSC) or Random Sequence Complexity (RSC) [42]. Functional Sequence Complexity (FSC) is a linear string of monomers or composite units that collectively perform some nontrivial function. Empirical evidence of the purely spontaneous formation of such strings, especially when more than 10 loci are involved, is sorely lacking. Ordinarily, FSC originates in association with PI as a material symbol system. FSC is usually a linear, digital, cybernetic string of tokens representing syntactic, semantic and pragmatic prescription. Each successive sign in the string is a representation of a decision-node specific selection for function. This can be accomplished as the selection of a token in a material symbol system, or as the setting of a series of configurable switches (e.g., “dip” switches). FSC is a succession of algorithmic selections leading to function. Selection, specification, or signification of certain “choices” in FSC sequences results only from nonrandom selection. These selections at successive decision nodes cannot be forced by deterministic cause-and-effect necessity. If they were, nearly all decision-node selections would be the same. They would be highly ordered (OSC). Moreover, the selections cannot be random (RSC). No sophisticated program has ever been observed to be written by successive coin flips where heads is “1” and tails is “0”.

FSC can be measured in “Fits” [119,120,121]. The change in FSC can be quantified during both nucleic acid and protein evolution [120]. Nonphysical formal Prescriptive Information (PI) is often instantiated (recorded) into physical FSC. The syntax of token (nucleotide) selections, for example, functions similar to the selection of the syntax of Scrabble game tokens to spell words, except that the syntax of nucleotides provides additional dimensions of PI besides the purely representational linear digital prescription using language symbols. Extensive post transcriptional and post translative editing also adds additional dimensions. The fact remains, however, that life depends upon instructions and control mechanisms. Those instructions (PI) are instantiated largely into nucleotide (token) sequences. Life is wholly dependent upon a formal Material Symbol System. While the tokens (nucleotides) are physical (like Scrabble blocks of wood), their physicality is not the issue, at least not when it comes to the functionality of their codon syntax. The codon table is formal, not physical.

Genetic cybernetics inspired Turing’s, von Neumann’s, and Wiener’s development of computer science [122,123,124,125,126,127,128]. The discrete nature of genes, their resortability, and the linear digital nature of their sequencing “on” Turing tape-like chromosomes were all very well appreciated and pondered long before Watson and Crick’s publication in 1953 of the details of DNA’s exact chemical structure. Turing was inspired not only by Gregor Mendel’s work in 1866, but by Darwin’s emphasis in 1859 of the difference between genotype and phenotype. Turing appreciated the mutability of genotype, in one sense independent of phenotype, that made evolution possible. Church, who taught Turing, also appreciated these aspects of life, as well as the formally algorithmic processing of genotype that alone made genetic control of phenotypic expression possible.

Genomic and epigenomic cybernetics cannot be explained by models that metaphysically pre-assume the all-sufficiency of mass-energy interactions and the chance and necessity of physicodynamics alone. Genetic and genomic algorithmic controls are fundamentally formal, not physical. Like other formalisms, they can be instantiated into a physical medium of retention and channel transmission using a material symbol system or dynamically-inert configurable switches.

David D’Onofrio [129], Donald Johnson [108], M.Conrad [130], Wang [131], Ramakrishnan and Bhalla [132], Yaakov Benenson [133,134] and many others have compared artificial cybernetic systems with life’s cybernetics. Say Ramakrishnan and Bhalla:

Just as complex electronic circuits are built from simple Boolean gates, diverse biological functions, including signal transduction, differentiation, and stress response, frequently uses biochemical switches as a functional module [132].

We speak loosely as though “bits” of information in computer programs represented specific integrated binary choice commitments made with intent at successive algorithmic decision nodes. Technically, such an algorithmic process cannot possibly be measured by bits (−log_2_ P) except in the sense of transmission engineering. Shannon [135,136] was interested in signal space, not in particular messages. Shannon *mathematics* deals only with averaged probabilistic combinatorics. *FSC requires a specification of which sequences work to accomplish a named function.*

Bits in a computer program measure only the number of binary choice *opportunities*. Bits do *not* measure or indicate *which specific choices* are made. Enumerating the specific choices that work is the very essence of gaining information (in the intuitive sense). When we buy a computer program, we are paying for sequences of integrated specific decision-node choice-commitments that we expect to work for us. The essence of the instruction is the enumeration of the sequence of *particular* choices. This necessity defines the very goal of genome projects.

Life depends upon literal objective genetic algorithms. Algorithms are *processes* or procedures that produce a needed result, whether it is computation or the products of biochemical pathways*.* Processes or procedures depend upon strings of decision-node selections that are anything but random. In addition, they are not “self-ordered” by redundant cause-and-effect necessity. Every successive nucleotide is a quaternary “switch setting”. Many nucleotide selections in the string are not critical. But, those switch-settings that do determine a certain protein folding, especially, are highly “meaningful”. Functional switch-setting sequences are produced only by uncoerced selection pressure. There is a cybernetic aspect of life processes that is directly analogous to that of computer programming. More attention should be focused on the reality and mechanisms of *selection at the decision-node level* of biological algorithms. This is the level of covalent bonding in primary structure. Environmental selection occurs at the level of post-computational halting. The fittest already-computed phenotype is selected.

## 6. Life Is Instructed and Controlled by Prescriptive Information (PI)

Prescriptive Information (PI) is a subset of intuitive or semantic (meaningful) information. Semantic information conveys meaningful and functional messages from a source to a destination (semiosis). Meaningful implies that the message can be understood and acted upon by a receiving agent at the destination—at the far end of a Shannon channel.

Adami rightly argues that information must always be *about* something [137]. “Aboutness” is a common focus of attention in trying to elucidate what makes information intuitive [138,139]. However, aboutness is always abstract, conceptual, and formal. Efforts to define aboutness in purely physical terms have frustrated bioinformationists for decades [31,32,33,34]. The difficulty of defining and understanding semantic information is especially acute in genetics [108,140]. Oyama points to the many problems trying to relate semantic information to cellular biology [141]. Some investigators attempt to deny that genes contain meaningful information and true instructions [142,143,144,145,146,147,148,149]. Their arguments strain credibility.

Intuitive, semantic information in biology is called Functional Information (FI) [150,151,152]. FI technically has two subsets: Descriptive (DI) and Prescriptive (PI) [79]. Unfortunately, many semantic information theorists make the mistake of thinking of functional information solely in terms of human epistemology, and specifically description (DI). This in effect limits the meaning of “function”. DI provides valued common-sense knowledge to human beings about the way things already are. *Being* can be described to provide one form of function. This subset of intuitive and semantic information, however, while highly functional, is very limited and grossly inadequate to address many forms of *instruction and control.*

Prescriptive information (PI) does far more than describe. Only PI provides “how to” information. PI instructs, steers and controls. We can thoroughly describe a new Mercedes automobile, providing a great deal of DI in the process. This functional DI, however, might tell us almost nothing about *how to design, engineer and build* that Mercedes. The term “functional information” as used in peer-reviewed naturalistic biological literature by Nobel laureate Jack Szostak *et al.* in 2003 [150,151,152] can be a completely inadequate descriptor of the “how to” information—the instructions—required to organize and program sophisticated utility. *Potential* formal function must be *prescribed* in advance by Prescriptive Information (PI) via decision node programming, not just described after the fact. As its name implies, PI specifically conceives and prescribes utility.

PI programs computational success in advance of halting. While it is true that halting must be empirically verified (the halting problem [122,153]), *computational success still must be prescribed in advance of its realization.* Selection pressure cannot do this (The GS Principle; see Section 12). PI either tells us what choices to make, or it is a recordation of wise choices already made [104]. When we install computer software, we are installing PI. Yet PI is not just limited to instruction. PI can also indirectly generate nontrivial computational success and cybernetic function in conjunction with external algorithmic processing. PI can be contained in the data stream and in the processing instructions.

PI can perform nonphysical “formal, nonphysical work”. PI arises from expedient choice commitments at bona fide decision nodes. The PI producing formal work can then be instantiated into physicality to marshal physical work out of nonphysical formal work [85,88].

Cybernetic programming is only one of many forms of PI. Ordinary language itself, various communicative symbol systems, logic theory, mathematics, rules of any kind, and all types of controlling and computational algorithms are forms of PI.

Empirical evidence of PI arising spontaneously from inanimate nature is sorely lacking [107,154]. Neither chance nor necessity has been shown to generate non-trivial PI [41,42,85,92,104,106,107,154,155,156]. Choice contingency, not chance contingency or law, prescribes nontrivial function. Choice contingency is a form of determinism. Determinism is not limited to physicodynamics. Choice contingency, when instantiated into physicality, can become a true cause of physical effects.

Selection of particular sequences of symbols (syntax) must follow prescribed arbitrary rules. It is only when these rules are followed by both sender and receiver that a meaningful/functional message can be successfully conveyed to its destination (semiosis) [108]. A meaning*less* message (a self-contradictory nonsense term) would fulfill no purpose and provide no functionality. It would therefore not qualify definitionally as a “message”. It would in fact be nothing more than a signal. Signals are not necessarily messages. A consistently repeating pulsar signal is not a meaningful message, and therefore not a message at all. Yet a pulsar signal contains high order and pattern.

It is common for non-specialists in biocybernetics and biosemiotics to try to define messages erroneously in terms of “order” or “patterns”. The patterns in the sand caused by wave action of the sea, for example, convey no meaningful message or cybernetic programming. Neither order nor patterns are the key to meaning, regulation, control or function. *Selection for potential function* at bona fide decision nodes and logic gates is. More conceptually complex PI is needed to compute and organize metabolism and life than is needed to generate our most advanced computer systems. Life is the most sophisticated of all integrated meta-systems.

PI is much more than intuitive semantic information. PI requires anticipation, “choice with intent,” and the diligent pursuit of Aristotle’s “final function” at successive bona fide decision nodes. PI either instructs or directly produces formal function at its destination through the use of controls, not mere constraints [85,86]. Once again, PI either tells us what choices to make, or it is a recordation of wise choices already made.

## 7. The Layers and Dimensions of Formal Biological PI Continue to Grow

The layers of biological PI continue to grow. Anti-sense transcription is occurring [157,158,159,160,161,162,163]. DNA is being read in both directions**. **Regulatory RNAs are often transcribed from the negative “anti-sense” strand that unwinds from the positive sense strand of DNA that prescribes proteins [157,164,165]. Linear digital prescription is bidirectional in DNA. Thus, the so-called “anti-sense” strand is full of sense and meaning. Genes overlap [157,166,167,168]. The efficacious nature of gene distribution as it relates to collective function is another form of PI. Chromatin coiling and its role in regulation, particularly in the simplest prokaryotes, is a form of PI [95,168,169,170]. Complementary strands can prescribe completely different functions, sometimes one strand regulating the coding function of the other. Multiple proteins are produced by one gene. Single proteins are being prescribed by sections from multiple genes. Widespread divergent transcription start sites (TSS) occur at protein-encoding gene promoters. Multi-protein complexes are far more extensive and crucial than expected. Supposed “junk” prescribes more biofunction than coding segments. The extent and kinds of editing, even post-translational editing, is mind-boggling. Noise-reducing Hamming block codes and bit parity prevent coding errors. Extraordinary repair mechanisms are in places that work around the clock as back up to preserve innumerable linear digital messages from corruption. Suicide (apoptosis) controls protect the greater good. The immune system, the one system that needs stochastic variation to protect against new and unexpected antigens, just happens to have it rather than the tight programming that most every other biological system depends upon. Three-dimensional conformation is far more liquid than expected, for good reason. The instructions prescribed by DNA are dynamic rather than static. Dynamic variation in coiling structure allows the same primary structure to prescribe widely variant functions.

All of this conceptual complexity does not obviate the importance of linear digital programming. It just adds to the sophistication and dimensions of the system’s overall PI. Micro RNA and regulatory protein sequences still have to be prescribed by linear digital discrete choices of specific nucleotides in their critical regions. Yes, many regions are not critical to a particular protein. Nevertheless, because of gene overlaps and the multi-dimensional nature of bioprescription, sequencing sections that may be irrelevant to one protein may be highly relevant to other proteins transcribed from the same gene.

## 8. Life Pursues Biofunctional Goals, and Succeeds

All known life is cybernetic [171,172,173,174]. This means that the integration and regulation of biochemical pathways and cycles into homeostatic metabolism is programmatically controlled, not just physicodynamically constrained. Life crosses *The Cybernetic Cut* [156] across a one-way CS (Configurable Switch) Bridge [156]. This bridge traverses a great ravine. On one side is found all those phenomena that can be explained by physicodynamics alone. On the other side are those phenomena than can be explained only by selection for potential (not-yet-existing) function. Traffic across this bridge flows only from the nonphysical world of formalism into the physical world through the instantiation of purposeful choices. Such instantiation requires arbitrary (dynamically inert) physical configurable switch-settings and selections of physical symbol vehicles in a material symbol system.

Except in pathologic states, life’s activities are almost always steered toward the formal goal of biochemical success. This includes the PI that instructs apoptosis for the greater good of the organism. The only system that seems to waste energy deliberately exploring randomness is the immune system. To prepare for exposure to an indefinite array of possible antigens, the immune system must be prepared to deal with any possible new combination of viral, bacterial, mycotic, or other parasitic invasion. The immune system is unique in its continuing perusal of potential genetic sequence space and three dimensional phase space. Every other biological system, however, expends energy with extraordinary efficiently to accomplish cooperative metabolic goals that are anything but random. Such pursuits are formal, not physicodynamic.

What about the generation of new genetic instructions? Isn’t duplication plus random variation the source of all new genetic Prescriptive Information (PI)? The answer is, “NO!” First, no new information exists in duplication, not even when Shannon uncertainty is confused with “information”. Second, no one has ever observed random variation generate non-trivial Prescriptive Information (PI) capable of generating or controlling new sophisticated function. Every supposed empirical support is trivial. Non-trivial illustrations are always theoretical rather than empirical or even rational. Non-trivial *vs.* trivial, of course, traverses a gray-scale of transition rather than being a black/white dichotomy. The issue of triviality cannot be resolved by appealing to statistical prohibitiveness or even to the Universal Plausibility Metric (UPM) and Universal Plausibility Principle (UPP) [175,176]. It is more a matter of *formal organizational extent* that currently cannot be quantified. The closest we can come might be to measure the number of “fits” (functional bits) that might be required to prescribe or organize a molecular machine capable of performing a needed function at a certain place and time [119,120,121]. The only point of excluding “non-trivial” function from the discussion is to prevent a cynic from pointing to some ridiculously minimal accidental “function” as supposed falsification of the larger principle.

The Universal Plausibility Metric [175,176] calculated for random generation of even a segment of the *Mycoplasma* genome consistently yields ξ values of <1. Given these measurements, The Universal Plausibility Principle [175,176] provides definitive scientific falsification of the chance hypothesis [140,177] that the genome of Mycoplasma was generated by duplication plus random variation. Even with a generation time of 20 minutes, the mutation rate, coupled with the low percentage of potentially beneficial mutations, does not provide sufficient opportunity for random variation of duplications to have prescribed such sophisticated genetic and genomic instructions. This is true even in a cosmic phase space over 14.5 billion years (only 10^18^ seconds, to be generous).

Any discussion of “duplication plus variation” should always elicit the question, “Duplication of what?” What is the source of the PI that is being duplicated? The duplication plus variation argument merely presupposes rather than explains the origin of any PI that might be duplicated. The question is, “Where did *any* PI come from in the first place?”

Contrary to public opinion, random mutations are not the modus operandi of genetics, genomics or epigenomics. Extensive error-preventing and error-correcting mechanisms are employed by life to protect the integrity of its existing PI against random variation (noise pollution of already programmed PI). The current Kuhnian paradigm rut notwithstanding, life makes no effort to pursue programming success through mutations. Instead, genomes contain an abundance of redundantly coded and duplicated information to protect it against mutations. Almost daily in the literature this redundancy is being shown to be purposeful, not the result of accumulated noise as originally thought. Empirical evidence is overwhelming that even life manifests no exemption from the normal decline in informational integrity as described by The OCD Law (The Law of Organizational and Cybernetic Decline) [84].

## 9. The Law of Organizational and Cybernetic Deterioration/Decline (The OCD Law)

The OCD Law [84] states that, absent the intervention of formal agency, any nontrivial organization or cybernetic/computational function instantiated into physicality (e.g., integrated circuits; programmed computational success, architectural and other engineering feats) will invariably deteriorate and fail through time. This deterioration may not be continual. However, it will be continuous (off and on, with overall downhill consistency through time). Computers, robots, all forms of Artificial Intelligence, Artificial Life, and even cellular life are subject to the OCD Law. Messages instantiated into material symbol systems or electronic impulses will invariably progress toward gibberish, dysfunction, and fail.

The OCD Law [84] should not be confused with the Second Law of Thermodynamics. The OCD Law is not concerned with the entropy of statistical mechanics or the “entropy” or “mutual entropy” of Shannon’s probabilistic combinatorial uncertainty. Heat exchange, heat dissipation, phase changes, order and disorder are not at issue. The OCD Law addresses only the *formal organization and utility* already instantiated into physical media and environments. Only purposeful choice contingency at bona fide decision nodes can rescue from eventual deterioration the organization and function previously programmed into physicality.

The Second Law of Thermodynamics works only on the mass/energy into which the formal instructions have been instantiated (recorded). When physical tokens, configurable switches and circuits deteriorate under the 2nd Law, the formal PI instantiated into these media tend to lose their physical recordation reliability. The Second Law, however, has no direct effect on nonphysical formalisms themselves that were instantiated—only the physical medium of recordation is affected. This in turn leads to the decline of instantiated cybernetic function.

The closely related Organization (O) Principle [84] states that nontrivial formal Organization can be produced *only* by Choice-Contingent Causation and Control (CCCC). ([84], Sec 9).

## 10. Choice-Contingent Causation and Control (CCCC)

CCCC is a decision-theory-based formalism defining how potential function and organization are achieved. CCCC is the only known cause of nontrivial function and organization. Under no circumstances can CCCC be explained or produced by chance and/or necessity. CCCC is the essence of any formalism. Evolution cannot produce or explain CCCC. Evolution has no goal, and cannot make programming choices at the molecular genetic level (The Genetic Selection (GS) Principle; see Section 12) [178,179]. Natural selection can only favor the best already-computed, already-living phenotypes. Life is controlled, not merely physicodynamically constrained, with pragmatic choices in pursuit of formal function. Neither physicodynamics nor evolution can make such programming choices at life’s logic gates and configurable switch settings.

As we used to teach decades ago, and still should, “Life is not ‘a bag of enzymes’.” Life is a concert of highly optimized and coordinated genetic algorithms. Metabolism and homeostasis far from equilibrium are impossible to achieve without collective and cooperative algorithmic optimizations [88]. Algorithmic optimization is a formal pursuit. It cannot be accomplished by cause-and-effect determinism. The cause-and-effect determinism of physicodynamics is not the only form of causation. The programming of PI and the successful computation it instructs can only be achieved via CCCC [84,85,107,180]. CCCC is ultimately far more important to any known formal system. CCCC steers events toward pragmatic results that are valued by agents. **CCCC** is a true primary cause leading to very real effects, particularly the effect of useful work rather than mere physicodynamic constraint. CCCC can generate extraordinary degrees of *unique functionality* that have never been observed to arise from randomness or law-described necessity. Neither physicodynamics nor evolution can pursue *potential* utility (e.g., the programming of computational success prior to its realization). CCCC does. CCCC is the only known cause and governor of formalisms.

## 11. Life Organizes Its Components into Sustained Functional Systems (SFS)

First, we must be clear on what is a bona fide “system”. A “system” is an abstract, conceptual organization generated by choice contingency, not chance or necessity, that typically generates formal processes or procedures with pragmatic results [82,83,88]. A “weather system” is not a true system. It is merely a physicodynamic interface of wind, temperature and atmospheric pressure differential. A weather front may involve phase changes and manifest self-ordering (e.g., a hurricane); but it is not organized. It manifests no choice contingency, no purposes or goals, no accomplishment of function or utility. Weather fronts have no formal components, no computational achievements, no algorithmic optimization, and no intended purpose.

Not even the simplest Sustained Functional Systems (SFS) [82,84,88] (e.g., the first non trivial heat engine) can be organized without the control of Maxwell’s demon (an agent) purposefully choosing when to open and close the trap door between compartments [88]. Inanimate nature will blindly produce equilibrium (by fixed, forced law) every time rather than the dichotomization and compartmentalization of faster moving inert gas particles from slower moving ones. No energy potential can be created from an inert gas absent the demon’s purposeful trap door choices. The demon’s trap door decisions are purely formal and nonphysical. They are not subject to the 2nd Law of thermodynamics. Only his physical sliding of the trap door up or down is subject to the 2nd Law. For the latter, usable energy for work must be generated, harnessed, stored, and called up when needed to move the trap door. Formal mechanisms must be organized and algorithmically optimized to accomplish paying the energy price of trap door sliding.

Maxwell’s Demon’s ability to generate a heat engine from *inert* gas molecules is critical to the discussion of life. Such a heat engine cannot be attributed to cause-and-effect physicochemical reactions, interactions or phase changes. Ideal gases are non-reactive. Thermodynamics alone is the issue in this model. The 2nd Law can only be temporarily and locally circumvented through Choice Contingent Causation and Control (CCCC) operating the trap door [1,2], not through the chance and necessity of physicodynamics. The same is true of metabolism and life. Controls, not constraints, make life possible. While life uses reactive chemistry rather than inert gas molecules, it must still algorithmically steer many thousands of events far from equilibrium to formally organize living “organisms”.

Some have argued that more work is needed to operate the trap door than what is accomplished by trap door operation. That may be true of a given individual work cycle. With wise programming of trap door operation, however, the effects of dichotomization of hot and cold particles can synergize. Formalisms have always been the key to the temporary and local circumvention of the 2nd Law. More efficient means of formally utilizing the stored energy can make the operation of the original trap door worthwhile. Generating the useful work to operate physically the trap door, however, requires the careful operation of even more formally controlled trap doors (logic gates and configurable switch settings in additional devices and machines). Life constructs and employs such molecular machines and sophisticated nanocomputers [108].

## 12. Life Is Governed by the Genetic Selection (GS) Principle

*The GS Principle* [92,178] states that *selection must occur at the molecular/genetic level*, not just at the fittest phenotypic/organismic level, to produce and explain life. In other words, selection for *potential* biofunction must occur upon formation of the rigid 3’5’ phosphodiester bonds in DNA and RNA sequences. This is the point at which functional linear digital polynucleotide syntax is prescribed. The selection of each nucleotide out of a phase space of four options constitutes the setting of a quaternary (four-way) configurable switch. The specific setting of these switches in nucleic acid primary structure (monomeric sequence) determines how translated biopolymer strings will fold into three-dimensional molecular machines.

Natural selection cannot operate at the genetic level. Selection pressure favors only *existing* biofunction. Even with existing function, natural selection does not select for isolated function over nonfunction. The inanimate environment could not care less whether anything functions. The environment has no preferences, values, goals or desires. Inanimate nature is blind and indifferent to *utility*. This is even truer of *potential* utility. Utility can only be defined, appreciated, and pursued formally, not physicodynamically. Pragmatics requires an added dimension beyond the four dimensions of Chance and Necessity [42,105,107,156].

Only the fittest already-living phenotypic organisms are secondarily “selected” by the environment, not abstract conceptual programming at decision nodes, logic gates, and configurable switch settings. Natural selection is nothing more than the differential survival and differential reproduction of the most successful already-living organisms. For an organism to be alive, it must first have many hundreds to thousands of biochemical pathways and cycles already integrated into holistic, cooperative, organized metabolic schemes. Few phenomena are more purposeful and goal-oriented than metabolism. Differential survival of the fittest species offers no model of mechanism for generating the cybernetic programming of linear digital genetic prescription. Biomessages provide linear digital instructions to prescribe cellular structures, specific transport and catalysis. Yet DNA is largely inert from a physichochemical standpoint. Natural selection cannot favor unrealized, not-yet-existent function represented in DNA syntax.

## 13. Life Is Organized, Not Self-Ordered

Self-ordering phenomena are not examples of self-organization. Self-ordering phenomena are simple, redundant, and low informational [42,106,107]. Self-ordered structures, whether sustained (e.g., crystals) or dissipative (e.g., the chaos theory first investigated by Prigogine) *contain no organization at all.*

Self-ordering events occur spontaneously daily. But, they do not involve decision nodes or dynamically-inert, purposeful, configurable switch settings. No logic gates need to be programmed with self-ordering phenomena. Self-ordering events involve no steering toward algorithmic success or “computational halting”. Self-ordering phenomena are purely physicodynamic and incapable of organizational attempts. Laws and fractals are both compression algorithms containing minimal complexity and information. Inanimate physicodynamics cannot exercise purposeful choices or pursue potential function. No model of undirected evolution pursues the goal of future utility.

Order cannot compute. Much life-origin literature appeals to “yet-to-be discovered laws of self-organization”. Laws, however, describe highly ordered/patterned behavior. Because they are parsimonious compression algorithms of data, they contain very little information. Given the high information content of life, expectation of a new law to explain sophisticated genetic algorithmic programming is ill founded. Considerable peer-reviewed published literature is erroneous because of failure to appreciate that the “complexity of life” could never arise from such highly “ordered,” low-informational physicodynamic patterning. Tremendous combinatorial uncertainty is required to record such exquisite PI. The complexity of life will never be explained by the highly ordered behavior that is reducible to the low-informational laws of physics and chemistry.

A crystal is highly ordered. Its description can be easily algorithmically compressed. A crystal is about as far from being “alive” as any physical state we could suggest. Every member of a 300-monomer string of adenosines (a homopolymer) can be specifically enumerated by stating: “Give me a set of adenosine molecules; repeatly connect one to another 300 times”. This is called a compression algorithm. The simplicity and shortness of this compression algorithm is a measure of the extremely low complexity and uncertainty of this polymer. Such a parsimonious statement of the full sequence is only possible because that sequence is so highly patterned. Such a highly ordered sequence lacks uncertainty, complexity, and the ability to instantiate PI. Such a parsimonious compression algorithm can enumerate each member of the 300-mer string with only fourteen words. This reality defines high order or pattern along with low information retaining potential.

The imagined spontaneous self-organization of ever-improving hypercycles [181,182,183,184,185], stoichiometric self-assemblies [186], and Ganti’s chemotons [187] have never been observed, let alone repeatedly observed. No prediction fulfillments have ever been realized. “Self-organization” provides no mechanism and offers no detailed verifiable explanatory power. The hypotheses of chemotons ever-growing capabilities are not even falsifiable. No lack of evidence or the repeated observation of hypercycle’s failure to arise is capable of providing falsification. So the notion is conveniently and indefinitely protected from any scientific challenge. It must just be accepted by blind faith. Any scientist who raises an eyebrow of healthy scientific skepticism is immediately labeled a heretic by the hierarchy from scientism’s presupposed imperative of metaphysical naturalism.

The mere presence of structure as opposed to heat-agitation-like molecular chaos tells us little about function and utility. Many rigid, sustained structures exhibit no function. In chaos theory, candle flames and tornadoes manifest seemingly sustained structure from a continual string of momentary self-ordered dissipative states. Neither kind of structure computes or optimizes any algorithmic function. None of Prigogine’s “dissipative structures” generates a Sustained Functional System (SFS) [88]. It is for good reason that Prigogine named them “dissipative”. Regardless of how long dissipative structures last, they certainly produce no sophisticated functions. Sustained Functional Systems (SFS’s) do.

## 14. Under No Circumstances Can Chaos Produce Life’s Ultra-Organization

The very definition of chaos is “a bounded state of disorganization that is extremely sensitive to the effects of initial conditions”. Disorganization cannot produce organization.

Note that chaos is a disorganized state of matter, *not a disordered* state of matter. A considerable amount of order can arise spontaneously out of chaos. This is what chaos theory is about. All we have to do to observe spontaneous self-ordering is to pull the stopper out of our bathtub drain. Water molecules quickly self-order into a swirl—a vortex—from purely physicodynamic complex causation. We mistakenly call this self-ordering “self-organization,” but the vortex is not in the least bit organized. It is only self-ordered [107]. What is the difference? No decision nodes are required for a bathtub swirl to self-order out of seemingly random Brownian motion. Proficient programming choices are not required for heat agitation of water molecules to self-order into a vortex. No configurable switches have to be purposefully set, each in a certain way, to achieve self-ordering. No pursuit of a goal is involved. No algorithmic optimization is required. In addition, Prigogine’s dissipative structures do not DO anything formally productive. They possess no ability to achieve computational success. They do not construct sophisticated Sustained Functional Systems (SFS) [88]. Dissipative structures are momentary. They only appear sustained (e.g., a candle flame) because of we observe through time a long string of momentary dissipative events or structures. This is where their name comes from. They cannot generate a sustained functional machine or system with optimized functionality.

Neither chaos nor the edge of chaos can produce a

(1) Calculus
(2) Algorithm
(3) Program that achieves computational success
(4) Organizer of formal function
(5) Bona fide system

Chaos is capable of producing incredibly complex physicodynamic behavior. We must never confuse this complexity with formal function, however. Order spontaneously appears out of disorder in the complete absence of any formal creative input or cybernetic management. But, no algorithmic organization is produced by a candle flame. What seems to be a totally random environment is in fact a caldron of complex interaction of multiple force fields. The complexity of interactive causation can create the illusion of randomness, or of very real self-ordering. There may also be as-of-yet undiscovered physical causes. But, dissipative structures self-order; they do NOT self-organize. The dissipative structures of chaos theory are unimaginative. Highly ordered structures contain very little information. Information retention in any physical medium requires freedom of selection of configurable switch settings. Switches must be “dynamically inert” with respect to their function to serve as logic gates.

The dissipative structures of chaos theory are

(1) Highly ordered
(2) Monotonous
(3) Predictable
(4) Regular (vortices, sand piles)
(5) Low informational
(6) Strings of momentary states

Dissipative structures are usually destructive, not cybernetically constructive (e.g., tornadoes, hurricanes). Trying to use “chaos” and “complexity” to provide mechanism for “self-organization” is like trying to use the Shannon transmission engineering to explain intuitive information, meaning and function.

Shannon’s equations define negative “uncertainty,” not positive “surprisal”. Functional “surprisal” requires the acquisition of positive specific semantic information. Just as we cannot explain and measure “intuitive information” using Shannon combinatorial uncertainty, we cannot explain a truly organized system appealing to nothing but a mystical “edge of chaos”. Reduced uncertainty (“mutual entropy”) in Shannon theory comes closer to semantic information. To achieve this, however, we have to mix in the formal elements of human knowledge gained by mathematical subtraction of “after uncertainty” from “before uncertainty”. We measure the reduced uncertainty of *our knowledge*. Prior background knowledge and agent processing of that knowledge is already at play. At that point, we are no longer talking about objective information in nature. We are only talking about human epistemology. Human consciousness is highly subjective. The second we insist on defining information solely in terms of a human observer and knower, we have destroyed all hope of elucidating the derivation of objective information in evolutionary history, especially at the intra-cellular or protobiont level.

The disorganization of chaos is characterized by conceptual uncertainty and confusion. Disorganization lacks sophisticated steering and control. Disorganization pursues no purpose. Even if chaos had purpose, it would lack all means of accomplishing purpose. If chaos by definition is a bounded state of disorganization, how could we possibly attribute self-organization to chaos? No scientific basis exists for granting formal capabilities to chaos, complexity or catastrophe. None of these three has ever been observed to produce formal integration and algorithmic organization of any kind.

Scientists accomplish impressive feats using the applied science of “nonlinear dynamics”. But, the capabilities of this applied science all-too-easily get confused with the capabilities of chaos itself. Chaos generates nothing close to formal function. We overlook the considerable degree of “investigator involvement” and artificial steering that goes into nonlinear dynamic experiments. Formal mathematics is invariably employed by agents to achieve some goal.

Three-dimensional conformation of molecular machines is largely determined by the minimum-free-energy sinks of primary structure folding. The primary structure of any protein or sRNA is the already-covalently-bound sequence of particular monomers that serve as configurable switch settings.

## 15. Life Accomplishes Intuitive (Functional) Work, Not Just the Physics’ Definition of Work

The physics’ definition of “work” has never done justice to the intuitive meaning of “work”.

The everyday meaning of “work” presupposes the accomplishment of some desired formal function, or the fulfillment of some perceived utilitarian need. The naturalization (materialization; physicalization) of science in an effort to avoid superstition and “vitalism,” fostered concepts of work in physics that are devoid of any purpose, formal functionality, or usefulness. In physics, “work” usually reduces to nothing more than heat transfer. Heat transfers occur daily in physicochemical interactions. They frequently have nothing to do with utility or life.

Any living cell is incredibly organized and goal-oriented toward the accomplishment of useful work. A living organism values and pursues staying alive, selfish advantage, and reproduction. To accomplish these goals, however, requires the active pursuit of thousands of subordinate formal goals. The cell must perform a large variety of molecular tasks that defined “useful work” long before Homo sapiens arrived on the scene to observe and ponder it.

Metabolism is the most highly integrated, holistic, conglomerate of organized formal functions known to science. How did life get so organized and goal-oriented out of an inanimate prebiotic environment that could care less about function or useful work? Chance and necessity cannot pursue function, let alone such an extraordinary degree of cooperative work.

The answer lies in the fact that life is formally programmed to pursue and perform such functions. It is not just blindly constrained by an indifferent physicodynamic cause-and-effect determinism. It is steered toward needed utility and formally controlled with the clear intent to stay alive and reproduce. Only a purely materialistic philosophic imperative precludes our acknowledgement of this obvious fact.

Non-trivial “useful work” is always associated with life. Life is the only known producer of useful work. Far more importantly, however, life itself depends upon even more sophisticated useful work at its molecular level. It employs magnificent molecular machines and nanocomputing to accomplish its formal goals [108]. Life could not be alive or stay alive without performing pragmatic work. Life is fundamentally formal, and only secondarily physical. The objective “facts of life” provide evidence for the reality and validity of The Formalism > Physicality (F > P) Principle [84].

The F > P Principle states that “Formalism not only describes, but preceded, prescribed, organized, and continues to govern and predict Physicality”. The F > P Principle is an axiom that defines the ontological primacy of formalism in a presumed objective reality that transcends both human epistemology, our sensation of physicality, and physicality itself. The F > P Principle works hand in hand with the Law of Physicodynamic Incompleteness [84], which states that physicochemical interactions are inadequate to explain the mathematical and formal nature of physical law relationships. Physicodynamics cannot generate formal processes and procedures leading to nontrivial function. Chance, necessity and mere constraints cannot steer, program or optimize algorithmic/computational success to provide desired nontrivial utility.

As a major corollary, physicodynamics cannot explain or generate life. Life is invariably cybernetic. The F > P Principle denies the notion of unity of Prescriptive Information (PI) with mass/energy. The F > P Principle distinguishes instantiation of formal choices into physicality from physicality itself. The arbitrary setting of configurable switches and the selection of symbols in any Material Symbol System (MSS) is physicodynamically indeterminate—decoupled from physicochemical determinism.

Naturalistic science tends merely to metaphysically presuppose initial disorganization in its various cosmologies and cosmogonies. Mass/Energy is allowed, but formal organization is usually seen to arise only from human consciousness. If organization in nature is allowed, it is confused with low-informational order and pattern, or is believed to have somehow miraculously created itself out of chance and necessity, a logical impossibility.

How was it determined that reality was initially chaotic, and only physical? Certainly not scientifically. The pre-assumption of ultimate chaos is not only purely metaphysical; it is antithetical to repeated observations of current reality, and to abundant formal prediction fulfillments of an underlying organization. The presumption of ultimate chaos is contrary to the logic theory upon which math and science are based. Overwhelming empirical evidence exists that reality is *not* fundamentally chaotic. Not only repeated observation, but innumerable fulfilled predictions of physical interactions based solely on mathematical models is far more suggestive that physicality unfolds according to formalism’s ultimate integration, organization and control of physicality.

## 16. Conclusions

What is the ingredient missing from inanimate physicodynamics that makes life possible? The answer is *formal control mechanisms.* This “regulation,” as it most often appears in the literature, is instructed by Prescriptive Information (PI) and its algorithmic processing—both elements being uniquely produced by life. In addition, both elements seem to have been inherent *in* life at the subcellular level in its earliest and simplest forms. Molecular biology itself is programmed, algorithmically processed, and purposefully regulated to achieve the highly integrated, formal biofunction we glibly call “metabolism” [108].

All of these phenomena are as nonphysical and formal as mathematics; and unique to life. We cannot just glibly write them off philosophically as being too “Cartesian”. Materialistic presuppositional commitments are causing us to turn our backs on a rapidly growing empirical biological reality that hollers into our deaf ears, “Materialism is dead!” We will never understand life under the purely metaphysical imperative, “Physicodynamics is all there is, ever was, or ever will be”. Professional philosophers of science rightly respond, “SEZ WHO?” How was that pontification scientifically determined? The scientific method itself cannot be reduced to mass and energy. Neither can language, translation, coding and decoding, mathematics, logic theory, programming, symbol systems, the integration of circuits, computation, categorizations, results tabulation, the drawing and discussion of conclusions. The prevailing Kuhnian paradigm rut of philosophic physicalism is obstructing scientific progress, biology in particular. There is more to life than chemistry. All known life is cybernetic. Control is choice-contingent and formal, not physicodynamic.
